# Complete mitogenome of the entomopathogenic fungus *Orbiocrella petchii*

**DOI:** 10.1080/23802359.2020.1787258

**Published:** 2020-07-09

**Authors:** Wei-Rong Wang, Yu-Xi Chen, Zhong Yan, Xiu-Yan Wei, Jun-Zhi Qiu, Yong-Jie Zhang

**Affiliations:** a School of Life Science, Shanxi University, Taiyuan , PR China; b State Key Laboratory of Ecological Pest Control for Fujian and Taiwan Crops, College of Life Sciences, Fujian Agriculture and Forestry University, Fuzhou, PR China

**Keywords:** *Orbiocrella petchii*, *Torrubiella petchii*, mitogenome, *Clavicipitaceae*

## Abstract

In this study, the complete mitogenome of an entomopathogenic fungus *Orbiocrella petchii* (syn. *Torrubiella petchii*) was assembled and annotated. This circular mitogenome was 23,794 bp in length and consisted of 2 *rRNA* genes (*rnl* and *rns*), 25 *tRNA* genes, and 14 standard protein-coding genes of the oxidative phosphorylation system. Two group I introns were identified, and they encoded ribosomal protein S3 (in *rnl*) or a GIY-YIG endonuclease (in *nad1*). Phylogenetic analysis based on mitochondrial DNA sequences confirms *O. petchii* in the family of *Clavicipitaceae*.

*Torrubiella* is characterized as arthropod pathogens that produce superficial perithecia on a loose mat of hyphae or a highly reduced non-stipitate stroma. *Torrubiella petchii* (currently *Orbiocrella petchii*) is a pathogen of scale insects found on bamboo leaves (Hywel-Jones 1997). This fungus was recently transferred to *Orbiocrella* (Johnson et al. 2009), which is currently a monospecific genus. This species has been shown to be a unique source of bioactive aromatic polyketides (Isaka et al. 2019); however, its studies at the molecular level is still very limited. Herein, we present the mitogenome of *O. petchii* SD3, which was isolated from a scale insect (Hemiptera) underside of a bamboo leaf in Ding Hu Mountain Nature Reserve, Guangdong Province, China (23°10′N, 112°31′E) and was deposited in the China General Microbiological Culture Collection Center (CGMCC), China (Accession 3.17637).

Total DNA of SD3 was randomly sheared to fragments of 400 bp followed by sequencing on an Illumina HiSeq 2500 platform in 2 × 250 bp reads. Mitogenome was *de novo* assembled from clean reads using NOVOPlasty (Dierckxsens et al. 2017) and then annotated as described previously (Zhang et al. 2017). Introns are named according to proposals suggested by Johansen and Haugen (2001) and Zhang and Zhang (2019).

The mitogenome of *O. petchii* SD3 (GenBank accession: MT447058) is a circular molecule of 23,794 bp with an AT content of 71.88%. This mitogenome encodes 2 ribosomal RNAs (*rnl* and *rns*), 25 tRNAs, and 14 conserved proteins of the oxidative phosphorylation system (*nad1-6*, *4 L*; *cob*; *cox1-3*, and *atp6*, *8*, *9*). These *tRNA* genes code for all 20 standard amino acids. There are three *tRNA* genes for methionine with the same anticodon, two *tRNA* genes for arginine, leucine, and serine with different anticodons. The majority of *tRNA* genes are clustered upstream (*trnV*, *I*, *S2*, *W*, *P*) and downstream (*trnT*, *E*, *M1*, *M2*, *L1*, *A*, *F*, *K*, *L2*, *Q*, *H*, *M3*) of the *rnl* gene, and downstream (*trnY*, *D*, *S1*, and *N*) of the *rns* gene. No intergenic free-standing open reading frame (ORF) is identified. For the two neighboring gene pairs commonly found in hypocrealean species, *nad3* follows immediately *nad2*, whereas *nad5* overlaps one nucleotide with its upstream gene *nad4L*. It should be noted that *nad6* overlaps 50 bp at the 3′ end with *trnV*. All protein-coding genes in the mitogenome start by ATG and terminate by TAA except *cox1* which terminates by TAG. This mitogenome is rather compact with genic regions (21,793 bp) accounting for 91.5% of the mitogenome. All genes are transcribed at the same strand.

Only two introns are identified, and they invade *nad1* and *rnl*. Both introns belong to the group I intron family but two specific subgroups, namely IB (nad1P636) and IA (mL2450). Intronic ORFs encode GIY-YIG endonuclease (in *nad1*) or ribosomal protein S3 (in *rnl*). Intronic regions (including intronic ORFs) have a total length of 2306 bp.

Phylogenetic analysis based on mitochondrial DNA sequences confirms *O. petchii* as a member of Clavicipitaceae, and it has a close relationship to the grass symbiont *Epichloe festucae* (Figure 1). This study supports the conclusion that *Torrubiella* is not monophyletic due to the fact that *Torrubiella petchii* (currently *O. petchii*) clusters in Clavicipitaceae and *Torrubiella confragosa* (currently *Akanthomyces lecanii*) clusters in Cordycipitaceae (Zhang et al. 2020).

**Figure 1.**
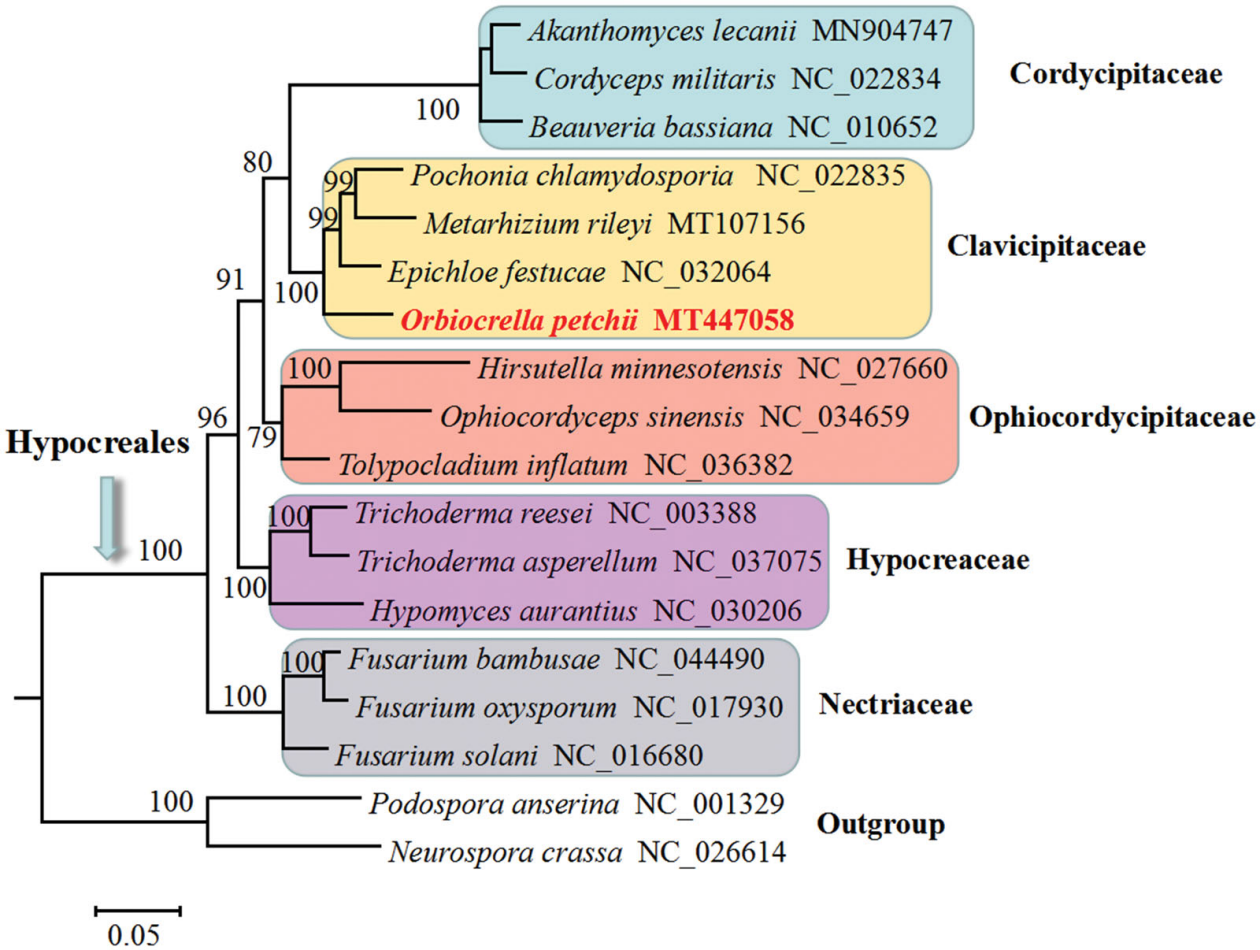
Phylogenetic analysis of Hypocreales species based on mitochondrial nucleotide sequences. We used three representative species of all families with available mitogenomes in *Hypocreales*. Two *Sordariales* species (*Podospora anserine* and *Neurospora crassa*) were used as outgroups. The whole mitogenome sequences (or exonic sequences in cases with alignment difficulties) of these species were aligned and trimmed using the HomBlocks pipeline (Bi et al. 2018), resulting in an alignment of 6667 characters. Phylogenetic reconstruction was performed using the maximum likelihood approach as implemented in RAxML version 8.2.12 (Stamatakis 2014). Support values were given for nodes that received bootstrap values ≥70%. GenBank accession numbers followed after fungal taxon names.

## Data Availability

The *O. petchii* SD3 mitogenome sequence was deposited in GenBank under accession number MT447058 (https://www.ncbi.nlm.nih.gov/nuccore/MT447058).
